# On One of the Causes of Death from Chloroform

**Published:** 1871-08

**Authors:** Andrew H. Smith

**Affiliations:** New York.


### ARTICLE V.

### *On one of the Causes of Death from Chloroform.*

Read before the New York Journal Association.

By Andrew H. Smith, M. D., of New York.

Mr. President: I will not absorb the time of the Society in an elaborate view of the different theories which have been advanced as to the modes in which chloroform produces death. The subject is a hackneyed one, and is doubtless familiar to all present. The facts involved are stated with great clearness and precision by Dr. Squibb, in a paper read before the State Dental Society at its last session. He states that the narcotism of chloroform may be symmetrical or asymmetrical. If symmetrical, it invades the different systems and organs in a regularly progressive order, the heart and respiratory organs resisting their influence the longest. But, if carried too far, even these may succumb, and death is the result. On the other hand, when asymmetrical, some organs are affected to a disproportionate degree, and if they be organs whose function is immediately necessary to life, as the respiratory muscles or the heart, a fatal result may take place before the general narcotism is developed.

It is generally stated that in much the larger proportion of cases, death results from cardiac syncope. Dr. Snow considers this to have been the case in all but six of fifty cases quoted by him in which the symptoms were more or less fully described. In nearly every instance, however, the only evidence that the heart had ceased to act was, that the pulse could not be felt at the wrist. But this test cannot be otherwise than fallacious, for it is a matter of daily observation that the heart may continue its action for some time after the radial pulse can no longer be perceived.

In two cases in which death from chloroform occurred under my observation, respiration ceased suddenly before unconsciousness had taken place, the action of the heart continuing for a considerable time. In several instances I have unintentionally destroyed animals with chloroform while practising vivisection, and in every case the pulsations of the heart continued after respiration had ceased. Dr. Snow seems to have observed this more frequently in animals than he is disposed to admit its occurrence in the human subject, but the numerous instances in which impending death is averted by artificial respiration seem to point to the breathing as the function first impaired in a considerable proportion of cases in man as well as in the lower animals.

But the object of this paper is to call attention to a mode of death which differs both from cardiac syncope and from paralysis of the respiratory muscles. I refer to *direct local anæsthesia of the lungs.*

It is well known that the movements of respiration, although to some extent under the influence of the will, are chiefly reflex in their character. This is shown by the fact that they continue during the unconsciousness of sleep, of anæsthesia, or of coma, and that beyond a certain point they are not under the control of the individual. Now, every reflex movement requires as its antecedent an impression upon a sensitive nerve. In this case the impression is chiefly upon the pulmonary nerves, and results from an excess of carbonic acid and a deficiency of oxygen in the blood. That the origin of the movements is peripheral and not central is shown by the fact that, that section of the pneumogastrics greatly reduces the frequency of the respiration, which would not be the case if the unaerated blood acted directly upon the respiratory centres as strychnia does upon the cord.

The character of the irritation which excites the movements of respiration may be appreciated by simply holding the breath. It is a sensation entirely peculiar, and is nature's demand for air just as hunger is nature's demand for food. By the French it is called the *besoin de respirer*, the necessity for breathing.

Now, in a certain proportion of cases, I believe that chloroform destroys life by its local anæsthetic effect upon the lung, rendering it insensible to the presence of carbonic acid, and thus removing the stimulus to respiration. The vapor of chloroform is a decided local anæsthetic, especially when applied to a mucous surface. It is well known that the senses of taste and smell are abolished at a very early stage of chloroform narcosis, and this effect is universally attributed to the local action of the chloroform. Dr. Squibb, in his paper already referred to, calls attention to this local anæsthetic effect upon the larynx as explaining the fact that the glottis does not close to prevent the passage of anæsthetic vapors as it does in the case of other irritating substances. Precisely this effect, extended to a large portion of the lung, may act, as I conceive, to prevent entirely the impression upon the nerves which is required to excite respiratory movements.

An analogous result is observed in opium narcosis, in which the sensibility of the lungs becomes more and more blunted, requiring a progressively-increasiug accumulation of carbonic acid to cause the necessary stimulas to the respiratory act. Hence, we find the respiration becoming slower and slower, until it finally ceases altogether. The only difference is that, in the case of the opium, this result is brought about secondarily through the medium of the circulation, and the lungs simply share in the general anæsthesia, while with chloroform we have, in addition, the local effect from the direct topical action of the vapor.

It is to be borne in mind that the lungs partake of the general anæsthetic effect of the chloroform to an exceptional extent, as the blood with which they are supplied is charged more highly with the anæsthetic than the blood in other portions of the body. Now, the experiments of M. Caze, of Strasbourg, show the anæsthetics act rather upon the peripheral nerves than upon the nerve-centres. He states that a limb can be protected against the influence of chloroform inhalation by merely compressing the main artery that supplies it. Immediately on removing the pressure and restoring the circulation, the limb becomes insensible. If this be true, the lungs are evidently exposed to a disproportionate action, since the nerves are bathed by blood containing a much larger proportion of the anæsthetic than is contained in the general circulation. If we now add to this the effect of which chloroform-vapor exerts upon the nerves by its direct local action independently of the circulation the surprise is that perfect insensibility is not the rule rather than the exception.

It occasionally happens in opium narcosis that the anæsthetic effect upon the lungs is developed out of proportion to the narcotic action upon the sensorium, so that the respiration will become alarmingly slow while the patient is still sufficiently conscious to quicken his breathing at the command of the physician. A case of this kind is described by Dr. J. C. Smith, in the *Medical Record* for January 2d: "Some five years ago I saw a case in which the result occurred from the use of chloroform. I was giving the chloroform very slowly, the object being merely to allay pain, when suddenly the patient ceased to breath, without having been at any time wholly unconscious. Very much alarmed, I was about to begin artificial respiration when I observed that the eyelids were trembling, and that the general expression of the countenance was not that of profound unconsciousness. Laying my hand roughly upon the shoulder of the patient, I said in a sharp, quick tone, '*Breathe*,' and to my great relief she responded with a full inspiration. But there seemed to be no disposition to repeat the effort voluntarily, and for several minutes each respiratory act was performed only at my command. I waited several times, as long as I dared to, to see whether she would not breathe spontaneously, and carried the experiment to the limit of what seemed to me justifiable. I do not assert that this would have proved a fatal case if left to itself, still less if the usual restorative treatment had been resorted to; on the contrary, I think it probable in this case, when the accumulation of carbonic acid in the blood had reached a certain point, the anaæsthesia of the lung would have been overcome and respiration would have been resumed. Still, the case differed so decidedly from those of momentary suspension of breathing which we often see when ether is employed, that I think the experiment would have been full of danger.

I may add that about two years ago I endeavored to determine by experiment what would be the effect of producing within the lungs the peculiar partial anæsthesia which results from the application of tincture of aconite to the surface. With this view I caused an animal to inhale for a moment the tincture of aconite in the form of spray, using Richardson's nebulizer, The respiration became at once very much embarrassed, and death resulted after a few hours. But I was unable to separate to my own satisfaction the local action upon the lungs from the general toxic effect of the drug, which may have been absorbed through the pulmonary membrane in quantity sufficient to produce death.

Death from pulmonary anæsthesia would be more likely to result from a large amount of vapor being inhaled at a single inspiration. This may readily occur when the chloroform is poured upon a towel folded in a number of thicknesses and already warmed by the breath of the patient and by contact with the face. If the towel is formed into a cone, the cone may be well filled with the almost pure chloroform vapor. When this is suddenly applied to the face of the patient, the first inspiration must include an undue proportion of the anæsthetic. Hence, an inhaler like that of Dr. Sayre, in which the chloroform is vaporized by the current of air drawn through it, would afford much greater safety.—*New York Medical Journal.*

---

